# Supplementary material for: Generalized measurement error: Intrinsic and incidental measurement error
Source: PLoS One. 2023 Jun 29;18(6):e0286680. doi: 10.1371/journal.pone.0286680 (PMC10309644; doi:10.1371/journal.pone.0286680)
Supplement: S1 File — (PDF) [file pone.0286680.s001.pdf]

S1 Appendix 1. Proof of Proposition 4.

Proof. Begin by expanding the sum of squares in (9) and then simplifying. (Note that we will make frequent use of the fact that we are assuming mutually independent measurements.) To whit:

$$\frac{1}{n-1}\sum_{i=1}^{n}\left[\int_{\mathbb{R}} x\ d\mu_{\omega_i}(x) - \overline{X}_\rho\right]^2 = \frac{1}{n-1}\sum_{i=1}^{n}\left(\int_{\mathbb{R}} x\ d\mu_{\omega_i}(x)\right)^2$$
$$- \frac{2}{(n-1)n}\sum_{i=1}^{n}\int_{\mathbb{R}} x\ d\mu_{\omega_i}(x)\sum_{j=1}^{n}\int_{\mathbb{R}} x\ d\mu_{\omega_j}(x) + \frac{1}{(n-1)n^2}\sum_{i=1}^{n}\left(\sum_{j=1}^{n}\int_{\mathbb{R}} x\ d\mu_{\omega_j}(x)\right)^2 \quad (28)$$

The second term in (9) is simply

$$\frac{1}{n}\sum_{i=1}^{n}\int_{\mathbb{R}} x^2\ d\mu_{\omega_i}(x) - \frac{1}{n}\sum_{i=1}^{n}\left(\int_{\mathbb{R}} x\ d\mu_{\omega_i}(x)\right)^2 \quad (29)$$

One can now combine the first term of the expansion in (28) with the second term in (29) and simplify to find

$$\frac{1}{(n-1)n}\sum_{i=1}^{n}\left(\int_{\mathbb{R}} x\ d\mu_{\omega_i}(x)\right)^2 - \frac{2}{(n-1)n}\sum_{i=1}^{n}\sum_{j=1}^{n}\int_{\mathbb{R}}\int_{\mathbb{R}} xy\ d\mu_{\omega_i}(x)d\mu_{\omega_j}(y)$$
$$+ \frac{1}{(n-1)n}\left(\sum_{i=1}^{n}\int_{\mathbb{R}} x\ d\mu_{\omega_i}(x)\right)^2 + \frac{1}{n}\sum_{i=1}^{n}\int_{\mathbb{R}} x^2\ d\mu_{\omega_i}(x) \quad (30)$$

Expanding the square in the third term of (30) and isolating the squared terms in the second term of (30) yields:

$$\frac{1}{(n-1)n}\sum_{i=1}^{n}\left(\int_{\mathbb{R}} x\ d\mu_{\omega_i}(x)\right)^2 - \frac{2}{(n-1)n}\int_{\mathbb{R}} x^2\ d\mu_{\omega_i}(x)$$
$$- \frac{4}{(n-1)n}\sum_{i\neq j}\int_{\mathbb{R}}\int_{\mathbb{R}} xy\ d\mu_{\omega_i}(x)d\mu_{\omega_j}(y) + \frac{1}{(n-1)n}\sum_{i=1}^{n}\left(\int_{\mathbb{R}} x\ d\mu_{\omega_i}(x)\right)^2$$
$$+ \frac{2}{(n-1)n}\sum_{i\neq j}\int_{\mathbb{R}}\int_{\mathbb{R}} xy\ d\mu_{\omega_i}(x)d\mu_{\omega_j}(y) + \frac{1}{n}\sum_{i=1}^{n}\int_{\mathbb{R}} x^2\ d\mu_{\omega_i}(x) \quad (31)$$

Now we can group the first and fourth terms, second and sixth terms, and third and fifth terms to find:

$$\frac{2}{(n-1)n}\sum_{i=1}^{n}\left(\int_{\mathbb{R}} x\ d\mu_{\omega_i}(x)\right)^2 - \frac{2}{(n-1)n}\sum_{i\neq j}\int_{\mathbb{R}}\int_{\mathbb{R}} xy\ d\mu_{\omega_i}(x)d\mu_{\omega_j}(y)$$
$$+ \frac{n-3}{(n-1)n}\sum_{i=1}^{n}\int_{\mathbb{R}} x^2\ d\mu_{\omega_i}(x) \quad (32)$$

We will now compare this expression with an expanded form of the AA-estimator AA-s_ρ^2 in (11). First, expand the square in (11):

$$\text{AA-}s_\rho^2 = \frac{1}{n-1}\sum_{i=1}^{n}\int_{\mathbb{R}^n} x_i^2 \; d\mu_{\otimes_n}(\boldsymbol{x}) - \frac{2}{n-1}\sum_{i=1}^{n}\int_{\mathbb{R}^n} x_i\overline{x} \; d\mu_{\otimes_n}(\boldsymbol{x}) + \frac{n}{n-1}\int_{\mathbb{R}^n} \overline{x}^2 \; d\mu_{\otimes_n}(\boldsymbol{x}) \quad (33)$$

The integral over the joint measure in the first term is the same as the integral over the marginal measures by independence of the measurement protocol. To generate crossterms for comparison with (32), we expand the factors of $\overline{x}$ in the last two terms above. For the second term of (33), we have

$$-\frac{2}{n-1}\sum_{i=1}^{n}\int_{\mathbb{R}^n} x_i\overline{x} \; d\mu_{\otimes_n}(\boldsymbol{x}) = -\frac{2}{(n-1)n}\int_{\mathbb{R}^n}\sum_{i=1}^{n}(x_i x_1 + \cdots x_i x_n) \; d\mu_{\otimes_n}(\boldsymbol{x}) \quad (34)$$

$$= -\frac{2}{(n-1)n}\left[\sum_{i=1}^{n}\int_{\mathbb{R}} x^2 \; d\mu_{\omega_i}(x) + 2\sum_{i\neq j}\int_{\mathbb{R}}\int_{\mathbb{R}} xy \; d\mu_{\omega_i}(x)d\mu_{\omega_j}(y)\right],$$

where we have again used independence to collapse the joint measure into the appropriate marginals. Similarly, for the third term of (33), we have

$$\frac{n}{n-1}\int_{\mathbb{R}^n}\overline{x}^2 \; d\mu_{\otimes_n}(\boldsymbol{x}) = \frac{1}{(n-1)n}\left[\sum_{i=1}^{n}\int_{\mathbb{R}} x^2 \; d\mu_{\omega_i}(x) + 2\sum_{i\neq j}\int_{\mathbb{R}}\int_{\mathbb{R}} xy \; d\mu_{\omega_i}(x)d\mu_{\omega_j}(y)\right] \quad (35)$$

Now we combine the first term of (33) with the first terms of (34) and (35), and combine the second terms of (34) and (35) to find:

$$\text{AA-}s_\rho^2 = \frac{n-1}{n^2}\sum_{i=1}^{n}\int_{\mathbb{R}} x^2 \; d\mu_{\omega_i}(x) - \frac{2}{(n-1)n}\sum_{i\neq j}\int_{\mathbb{R}}\int_{\mathbb{R}} xy \; d\mu_{\omega_i}(x)d\mu_{\omega_j}(y) \quad (36)$$

The second term here is identical to the second term of (32), while the first term of (36) is of the same form as the third term of (32). Asymptotically, these terms are equivalent, as they both behave like $\frac{1}{n}\sum_{i=1}^{n}\int_{\mathbb{R}} x^2 \; d\mu_{\omega_i}(x)$ for even moderately small n. Finally, the remaining first term in (32) is negligible if one assumes the RVVMs have uniformly bounded variances. This follows by an application of Jensen's Inequality. This assumption seems to be an eminently reasonable one, as it is hard to imagine any kind of informative measurement protocol that generates sample RVVMs with increasingly unbounded variances (i.e., greater uncertainty with successive measurements). □

Derivations of theoretical attenuation factors in Table 2 for Ex. 9.

For classic deterministic measurement error (i.e., trivial RVVMs), here is a demonstration of the classical results that a predictor X polluted by Berkson calibrated errors does not attenuate the OLS estimate of the simple regression slope towards the null, but one polluted by classically calibrated errors does.

Let $X^* = X + E$ (the generic measurement model), $Y = \beta X + \varepsilon$ (the population relationship of interest), and $Y = \beta^* X^* + \delta$ (the only relationship that can be empirically studied). Note that to simplify this presentation we have assumed without loss of generality that all intercepts are zero. As classically assumed, the errors E, ε, and δ are all independent of each other.

Assuming classical calibration, one has $\mathbb{E}(X^*|X) = X$, which implies $\mathbb{E}(E|X) = 0$. Then

$$\begin{aligned}
\beta^* &= \frac{\mathrm{Cov}(Y, X^*)}{\mathrm{Var}(X^*)} \\
&= \frac{\mathrm{Cov}(Y, X) + \mathrm{Cov}(Y, E)}{\mathrm{Var}(X) + \mathrm{Var}(E) + 2\mathrm{Cov}(X, E)} \\
&= \frac{\mathrm{Cov}(Y, X)}{\mathrm{Var}(X) + \mathrm{Var}(E)} \\
&= \beta \cdot \frac{\mathrm{Var}(X)}{\mathrm{Var}(X) + \mathrm{Var}(E)},
\end{aligned}$$

because $\mathrm{Cov}(X, E) = \mathbb{E}(XE) - \mathbb{E}(X)\mathbb{E}(E) = \mathbb{E}[\mathbb{E}(XE \mid X)] = 0$. The multiplier

$$AF := \frac{\mathrm{Var}(X)}{\mathrm{Var}(X) + \mathrm{Var}(E)}$$

is called the *attenuation factor* for the simple regression slope. In the presence of classical measurement error, this quantity is clearly less than 1, as for measurement protocol 3.

However, in the presence of Berkson measurement error (again, assuming deterministic measurements), there is no attenuation towards the null; i.e., AF = 1. To see this, note that we assume the same setup as before, except now $X^* = X + E$ with the calibrating condition $\mathbb{E}(X|X^*) = X^*$, which implies $\mathbb{E}(E|X^*) = 0$. Then

$$\begin{aligned}
\beta^* &= \frac{\mathrm{Cov}(Y, X^*)}{\mathrm{Var}(X^*)} \\
&= \frac{\beta\mathrm{Cov}(X, X^*) + \mathrm{Cov}(\varepsilon, X^*)}{\mathrm{Var}(X^*)} \\
&= \beta \cdot \frac{\mathrm{Cov}(X^*, X)}{\mathrm{Var}(X^*)} \\
&= \beta \cdot \frac{\mathbb{E}[\mathbb{E}(XX^* \mid X^*)] - \mathbb{E}(X)\mathbb{E}(X^*)}{\mathrm{Var}(X^*)} \\
&= \beta \cdot \frac{\mathbb{E}(X^{*2}) - \mathbb{E}(X^*)^2}{\mathrm{Var}(X)} = \beta.
\end{aligned}$$

Now we consider the situation for nontrivial measurement protocols. Since we are concerned with AA-estimators, we need to introduce notation for an arbitrary arrangement given a measurement protocol ρ for X. Define an arbitrary arrangement as $\{X^*(\omega) : \omega \in \Omega\} \subset \mathbb{R}^{\Omega}$. Then one can define $X^*(\omega) = X(\omega) + E(\omega)$ pointwise. By definition then, one has

$$AA(\beta^*) = \int_{\mathbb{R}^n} \frac{\text{Cov}(Y, X^*)}{\text{Var}(X^*)} \, d\mu_{\otimes_n}(X^*), \tag{37}$$

using (10). Notice that for all of our measurement protocols $Y \perp E$. Therefore, one can rewrite (37) as

$$\begin{aligned} AA(\beta^*) &= \int_{\mathbb{R}^n} \frac{\text{Cov}(Y, X)}{\text{Var}(X^*)} \, d\mu_{\otimes_n}(X^*) \\ &= \int_{\mathbb{R}^N} \beta \cdot \frac{\text{Var}(X)}{\text{Var}(X^*)} \, d\mu_{\otimes_n}(X^*). \end{aligned} \tag{38}$$

The multiplier of β is the attenuation factor. We now simplify this expression for each of the nontrivial measurement protocols 4 through 11.

First, note that $\text{Var}(X) = 9$ by definition. Thus, we only need to compute $\text{Var}(X^*)$ for the various measurement protocols. We have $\rho_4(\omega) = N(X(\omega), \sqrt{v(\omega)})$ where $X \sim N(5, SD = 3)$ and $v \sim U(0, 4)$, and $\tau_4(\omega) = \text{id}_Y(\omega)$. Therefore, $X^*(\omega) \sim N(X(\omega), \sqrt{v(\omega)})$, which implies that $E(\omega) \sim N(0, \sqrt{v(\omega)})$. So,

$$\begin{aligned} \text{Var}(X^*) &= \text{Var}(X) + \text{Var}(E) \\ &= 9 + \text{Var}[\mathbb{E}(E|v)] + \mathbb{E}[\text{Var}(E|v)]. \end{aligned}$$

Since $v \sim U(0, 4)$, we find $\text{Var}[\mathbb{E}(E|v)] + \mathbb{E}[\text{Var}(E|v)] = 0 + 2$. Therefore, $AF_4 = \frac{9}{9+2} = 0.818$.

Similarly, we have $\rho_5(\omega) = N(Z(\omega), \sqrt{v(\omega)})$ where $X \sim N(5, SD = 3)$, $v \sim U(0, 4)$, and $Z(\omega) \sim N(X(\omega), 1)$. Therefore, one can write $X^*(\omega) = X(\omega) + D(\omega) + E(\omega)$, where $E(\omega) \sim N(0, v(\omega))$, $D(\omega) \sim N(0, 1)$, and $X \perp E, D$. Thus, $\text{Var}(X^*) = \text{Var}(X) + \text{Var}(E) + \text{Var}(D) = 9 + 2 + 1$, and so $AF_5 = \frac{9}{9+2+1} = 0.750$.

The remaining calculations follow the same pattern; i.e., one needs only to determine $\text{Var}(X^*) - \text{Var}(X)$. For ρ_6, the RVVMs are always uniform over an interval of support with width equal to $2v(\omega)$. Therefore, $\text{Var}[\mathbb{E}(E|v)] + \mathbb{E}[\text{Var}(E|v)] = 0 + \mathbb{E}(4v^2/12) = \frac{4}{12}\mathbb{E}(v^2) = \frac{16}{9}$, and so $AF_6 = \frac{9}{9+16/9} = 0.835$. For ρ_7, the RVVMs are always uniform over an interval of support with width equal to $l_{max} - l_{min} = r_{max} - r_{min}$. Consequently, $AF_7 = \frac{9}{9+(l_{max}-l_{min})^2/12} = 0.733$. For ρ_8 and ρ_{10}, the RVVMs are always uniform over an interval of support with width equal to $4v(\omega)$. Therefore, $\text{Var}[\mathbb{E}(E|v)] + \mathbb{E}[\text{Var}(E|v)] = 0 + \mathbb{E}(16v^2/12) = \frac{1}{6}12\mathbb{E}(v^2) = \frac{256}{36}$, and so $AF_6 = \frac{9}{9+256/36} = 0.559$. For ρ_9, combining the previous derivation with the one for ρ_5 yields $AF_7 = \frac{9}{9+256/36+1} = 0.526$. Finally, since ρ_{11} is trivial and free of measurement error, the attenuation factor in (38) is exactly 1.
