# Supplementary material for: Generalized measurement error: Intrinsic and incidental measurement error
Source: PLoS One. 2023 Jun 29;18(6):e0286680. doi: 10.1371/journal.pone.0286680 (PMC10309644; doi:10.1371/journal.pone.0286680)
Supplement: S2 File — (PDF) [file pone.0286680.s002.pdf]

S2 Appendix 2. R code for in-text simulations.

Note: This code is also available on GitHub at: `https://github.com/edkroc/Supplemental_information_S2_PLOSOne_GeneralizedMeasurementError2023`

R code for simulation comparing the unbiased S_ρ^2 estimator of the variance with the AA-estimator of s_ρ^2, from the paragraph after Proposition 4:

```
M <- 100
s1 <- c(rep(NA,M))
s2 <- c(rep(NA,M))

for(k in 1:M){
N <- 10000
s <- c(rep(NA,N))
n <- 30
m <- rnorm(n,2,20)
v <- runif(n,0,5)
x <- c(rep(NA,n))

for(i in 1:N){
        for(j in 1:n){
                x[j] <- rnorm(1,m[j],sqrt(v[j]))
        }
        s[i] <- var(x)
}
s1[k] <- mean(s)

mrho <- sum(m)/n
srho1 <- c(rep(NA,n))
srho2 <- c(rep(NA,n))
for(j in 1:n){
        srho1[j] <- (m[j] - mrho)^2
        srho2[j] <- v[j]
}
srho <- sum(srho1)/(n-1) + sum(srho2)/n
s2[k] <- srho
}
s1-s2
mean(s1-s2)
```

Empirical estimation of the AA-estimators of the OLS solutions to the regression model (16) and their sampling, measuring, and total variances under measurement protocols 1 through 11 from Example 9.

```
##Set seed to generate sample data:
set.seed(9870)

##Number of bootstraps:
M <- 200

##Number of Monte Carlo steps:
N <- 2000

##Sample size:
n <- 100

#Set range of parameters for RVVMs:
v.min <- 0
v.max <- 4

##Define the parameters for the different measurement protocols:
x.omega <- rnorm(n,5,3)
v.omega <- runif(n,v.min,v.max)
w.omega <- runif(n,v.min,v.max)
x.l.trunc.mean <- 5 + 3*((-1)/(sqrt(2*pi)*0.5))
x.r.trunc.mean <- 5 + 3*(1/(sqrt(2*pi)*0.5))
x.trunc.var <- 9*(1-(1/(sqrt(2*pi)*.5))^2)
dl <- 10 - 12/sqrt(2*pi)
x.rho4.lmax <- (4*dl + sqrt( 16*dl^2 - 4*4*(dl^2 - 12*9*(1-2/pi)) ))/8
x.rho4.lmin <- dl - x.rho4.lmax
dr <- 10 + 12/sqrt(2*pi)
x.rho4.rmax <- (4*dr + sqrt( 16*dr^2 - 4*4*(dr^2 - 12*9*(1-2/pi)) ))/8
x.rho4.rmin <- dr - x.rho4.rmax

##Define the sample responses y as a function of x for the
##sample (doubles as data for first measurement protocol,
##free of intrinsic measurement error):
y.omega <- 1 + 5*x.omega + rnorm(n,0,.5)

##Set seed for Monte Carlo and bootstrapping:
#set.seed(242)
```

```
##Monte Carlo approximation to the AA-estimators for the
##regression model using the various nontrivial measurement protocols:
x.rho1 <- c(rep(NA,n))
coef1 <- matrix(c(rep(NA,2*N)),nrow=N,byrow=T)
x.rho2 <- c(rep(NA,n))
coef2 <- matrix(c(rep(NA,2*N)),nrow=N,byrow=T)
x.rho3 <- c(rep(NA,n))
coef3 <- matrix(c(rep(NA,2*N)),nrow=N,byrow=T)
x.rho4 <- c(rep(NA,n))
coef4 <- matrix(c(rep(NA,2*N)),nrow=N,byrow=T)
x.rho6 <- c(rep(NA,n))
coef6 <- matrix(c(rep(NA,2*N)),nrow=N,byrow=T)
x.rho7 <- c(rep(NA,n))
x.rho7mu <- c(rep(NA,n))
coef7 <- matrix(c(rep(NA,2*N)),nrow=N,byrow=T)
y.rho1 <- c(rep(NA,n))
coef8 <- matrix(c(rep(NA,2*N)),nrow=N,byrow=T)
coef9 <- matrix(c(rep(NA,2*N)),nrow=N,byrow=T)

for(j in 1:N){
        for(i in 1:n){
##x.rho1: First order Berkson and classical calibration:
                x.rho1[i] <- rnorm(1,x.omega[i],sqrt(v.omega[i]))
##x.rho2: First order classical calibration only:
                x.rho2[i] <- rnorm(1,rnorm(1,x.omega[i],1),
                                        sqrt(v.omega[i]))
##x.rho3: First order Berkson calibration only:
                if(x.omega[i] <= 5){x.rho3[i] <-
runif(1,x.l.trunc.mean-v.omega[i], x.l.trunc.mean+v.omega[i])}
                else if(x.omega[i] > 5){x.rho3[i] <-
runif(1,x.r.trunc.mean-v.omega[i], x.r.trunc.mean+v.omega[i])}
##x.rho4: First and second order Berkson calibrated,
##no classical calibration:
                if(x.omega[i]<=5){x.rho4[i] <-
runif(1,x.rho4.lmin, x.rho4.lmax)}
                else if(x.omega[i] > 5){x.rho4[i] <-
runif(1,x.rho4.rmin, x.rho4.rmax)}
##x.rho6: Berkson and classical calibration, more measuring variance:
                x.rho6[i] <- runif(1,x.omega[i]-2*v.omega[i],
x.omega[i]+2*v.omega[i])
##x.rho7: Classical calibration only, more measuring variance:
                x.rho7mu[i] <- rnorm(1,x.omega[i],1)
                x.rho7[i] <- runif(1,x.rho7mu[i]-2*v.omega[i],
x.rho7mu[i]+2*v.omega[i])
```

```
##y.rho1: Berkson and classical calibration in the response:
                y.rho1[i] <- rnorm(1,y.omega[i],10*sqrt(w.omega[i]))
        }
        mod1 <- lm(y.omega ~ x.rho1)
        coef1[j,] <- as.vector(summary(mod1)$coefficients[,1])
        mod2 <- lm(y.omega ~ x.rho2)
        coef2[j,] <- as.vector(summary(mod2)$coefficients[,1])
        mod3 <- lm(y.omega ~ x.rho3)
        coef3[j,] <- as.vector(summary(mod3)$coefficients[,1])
        mod4 <- lm(y.omega ~ x.rho4)
        coef4[j,] <- as.vector(summary(mod4)$coefficients[,1])
        mod6 <- lm(y.omega ~ x.rho6)
        coef6[j,] <- as.vector(summary(mod6)$coefficients[,1])
        mod7 <- lm(y.omega ~ x.rho7)
        coef7[j,] <- as.vector(summary(mod7)$coefficients[,1])
        mod8 <- lm(y.rho1 ~ x.rho6)
        coef8[j,] <- as.vector(summary(mod8)$coefficients[,1])
##Berkson and classical calibration in the response, no measurement
##error in predictor:
        mod9 <- lm(y.rho1 ~ x.omega)
        coef9[j,] <- as.vector(summary(mod9)$coefficients[,1])
}
aa.est1 <- apply(coef1,2,mean)
aa.est2 <- apply(coef2,2,mean)
aa.est3 <- apply(coef3,2,mean)
aa.est4 <- apply(coef4,2,mean)
aa.est6 <- apply(coef6,2,mean)
aa.est7 <- apply(coef7,2,mean)
aa.est8 <- apply(coef8,2,mean)
aa.est9 <- apply(coef9,2,mean)

aa.est1
aa.est2
aa.est3
aa.est4
aa.est6
aa.est7
aa.est8
aa.est9

##Bootstrapping and Monte Carlo approximation to the total
##variance of the sample regression coefficients:

aa.ests1 <- matrix(c(rep(NA,2*M)),nrow=M,byrow=T)
```

```
aa.ests2 <- matrix(c(rep(NA,2*M)),nrow=M,byrow=T)
aa.ests3 <- matrix(c(rep(NA,2*M)),nrow=M,byrow=T)
aa.ests4 <- matrix(c(rep(NA,2*M)),nrow=M,byrow=T)
aa.ests5 <- matrix(c(rep(NA,2*M)),nrow=M,byrow=T)
aa.ests6 <- matrix(c(rep(NA,2*M)),nrow=M,byrow=T)
aa.ests7 <- matrix(c(rep(NA,2*M)),nrow=M,byrow=T)
aa.ests8 <- matrix(c(rep(NA,2*M)),nrow=M,byrow=T)
mvar.ests1 <- matrix(c(rep(NA,2*M)),nrow=M,byrow=T)
mvar.ests2 <- matrix(c(rep(NA,2*M)),nrow=M,byrow=T)
mvar.ests3 <- matrix(c(rep(NA,2*M)),nrow=M,byrow=T)
mvar.ests4 <- matrix(c(rep(NA,2*M)),nrow=M,byrow=T)
mvar.ests5 <- matrix(c(rep(NA,2*M)),nrow=M,byrow=T)
mvar.ests6 <- matrix(c(rep(NA,2*M)),nrow=M,byrow=T)
mvar.ests7 <- matrix(c(rep(NA,2*M)),nrow=M,byrow=T)
mvar.ests8 <- matrix(c(rep(NA,2*M)),nrow=M,byrow=T)


for(k in 1:M){
        ##Index for bootstrapping:
        ind <- sample(c(1:n),n,replace=T)

        ##Create bootstrapped dataset:
        x <- c(rep(NA,n))
        v <- c(rep(NA,n))
        w <- c(rep(NA,n))
        y <- c(rep(NA,n))
        for(i in 1:n){
                x[i] <- x.omega[ind[i]]
                v[i] <- v.omega[ind[i]]
                w[i] <- w.omega[ind[i]]
                y[i] <- y.omega[ind[i]]
        }

        ##Monte Carlo approximation on the bootstrapped datasets:
        x.rho1 <- c(rep(NA,n))
        x.rho2 <- c(rep(NA,n))
        x.rho3 <- c(rep(NA,n))
        x.rho4 <- c(rep(NA,n))
        x.rho6 <- c(rep(NA,n))
        x.rho7 <- c(rep(NA,n))
        y.rho1 <- c(rep(NA,n))
        coef1 <- matrix(c(rep(NA,2*N)),nrow=N,byrow=T)
        coef2 <- matrix(c(rep(NA,2*N)),nrow=N,byrow=T)
        coef3 <- matrix(c(rep(NA,2*N)),nrow=N,byrow=T)
```

```
coef4 <- matrix(c(rep(NA,2*N)),nrow=N,byrow=T)
coef5 <- matrix(c(rep(NA,2*N)),nrow=N,byrow=T)
coef6 <- matrix(c(rep(NA,2*N)),nrow=N,byrow=T)
coef7 <- matrix(c(rep(NA,2*N)),nrow=N,byrow=T)
coef8 <- matrix(c(rep(NA,2*N)),nrow=N,byrow=T)
for(j in 1:N){
        for(i in 1:n){
                x.rho1[i] <- rnorm(1,x[i],sqrt(v[i]))
                x.rho2[i] <- rnorm(1,rnorm(1,x[i],1), sqrt(v[i]))
                if(x[i]<=5){x.rho3[i] <-
runif(1,x.l.trunc.mean-v[i], x.l.trunc.mean+v[i]) }
                else if(x[i]>5){x.rho3[i] <-
runif(1,x.r.trunc.mean-v[i], x.r.trunc.mean+v[i]) }
                if(x[i]<=5){x.rho4[i] <-
runif(1,x.rho4.lmin, x.rho4.lmax)}
                else if(x[i]>5){x.rho4[i] <-
runif(1,x.rho4.rmin, x.rho4.rmax)}
                x.rho6[i] <- runif(1,x[i]-2*v[i],x[i]+2*v[i])
                x.rho7mu[i] <- rnorm(1,x[i],1)
                x.rho7[i] <- runif(1,x.rho7mu[i]-2*v[i],
x.rho7mu[i]+2*v[i])
                y.rho1[i] <- rnorm(1,y[i],10*sqrt(w[i]))
        }
        mod1 <- lm(y ~ x.rho1)
        coef1[j,] <-
                as.vector(summary(mod1)$coefficients[,1])
        mod2 <- lm(y ~ x.rho2)
        coef2[j,] <-
                as.vector(summary(mod2)$coefficients[,1])
        mod3 <- lm(y ~ x.rho3)
        coef3[j,] <-
                as.vector(summary(mod3)$coefficients[,1])
        mod4 <- lm(y ~ x.rho4)
        coef4[j,] <-
                as.vector(summary(mod4)$coefficients[,1])
        mod5 <- lm(y ~ x.rho6)
        coef5[j,] <-
                as.vector(summary(mod5)$coefficients[,1])
        mod6 <- lm(y ~ x.rho7)
        coef6[j,] <-
                as.vector(summary(mod6)$coefficients[,1])
        mod7 <- lm(y.rho1 ~ x.rho6)
        coef7[j,] <-
                as.vector(summary(mod7)$coefficients[,1])
```

```
            mod8 <- lm(y.rho1 ~ x)
            coef8[j,] <-
                  as.vector(summary(mod8)$coefficients[,1])
      }

      ##Get AA-estimators of regression coefficients for each
      ##bootstrapped dataset:
      aa.ests1[k,] <- apply(coef1,2,mean)
      aa.ests2[k,] <- apply(coef2,2,mean)
      aa.ests3[k,] <- apply(coef3,2,mean)
      aa.ests4[k,] <- apply(coef4,2,mean)
      aa.ests5[k,] <- apply(coef5,2,mean)
      aa.ests6[k,] <- apply(coef6,2,mean)
      aa.ests7[k,] <- apply(coef7,2,mean)
      aa.ests8[k,] <- apply(coef8,2,mean)

      ##Get measurement variance of estimated regression
      ##coefficients for each bootstrapped dataset:
      mvar.ests1[k,] <- apply(coef1,2,var)
      mvar.ests2[k,] <- apply(coef2,2,var)
      mvar.ests3[k,] <- apply(coef3,2,var)
      mvar.ests4[k,] <- apply(coef4,2,var)
      mvar.ests5[k,] <- apply(coef5,2,var)
      mvar.ests6[k,] <- apply(coef6,2,var)
      mvar.ests7[k,] <- apply(coef7,2,var)
      mvar.ests8[k,] <- apply(coef8,2,var)

      print(k)
}

##Monte Carlo approximation of sampling variance of the
##AA-estimators using bootstrapped estimates:
s.var1 <- apply(aa.ests1,2,var)
s.var2 <- apply(aa.ests2,2,var)
s.var3 <- apply(aa.ests3,2,var)
s.var4 <- apply(aa.ests4,2,var)
s.var5 <- apply(aa.ests5,2,var)
s.var6 <- apply(aa.ests6,2,var)
s.var7 <- apply(aa.ests7,2,var)
s.var8 <- apply(aa.ests8,2,var)

##Monte Carlo approximation of measuring variance using
##bootstrapped estimates:
m.var1 <- apply(mvar.ests1,2,mean)
```

```
m.var2 <- apply(mvar.ests2,2,mean)
m.var3 <- apply(mvar.ests3,2,mean)
m.var4 <- apply(mvar.ests4,2,mean)
m.var5 <- apply(mvar.ests5,2,mean)
m.var6 <- apply(mvar.ests6,2,mean)
m.var7 <- apply(mvar.ests7,2,mean)
m.var8 <- apply(mvar.ests8,2,mean)

##Total standard error of OLS estimates of regression
##coefficients:
t.se1 <- sqrt(s.var1 + m.var1)
t.se2 <- sqrt(s.var2 + m.var2)
t.se3 <- sqrt(s.var3 + m.var3)
t.se4 <- sqrt(s.var4 + m.var4)
t.se5 <- sqrt(s.var5 + m.var5)
t.se6 <- sqrt(s.var6 + m.var6)
t.se7 <- sqrt(s.var7 + m.var7)
t.se8 <- sqrt(s.var8 + m.var8)

##Print results:
output <- list(list(aa.est1,aa.est2,aa.est3,aa.est4,aa.est6,aa.est7,
        aa.est8,aa.est9),
        list(t.se1,t.se2,t.se3,t.se4,t.se5,t.se6,t.se7,t.se8),
        list(sqrt(s.var1),sqrt(s.var2),sqrt(s.var3),sqrt(s.var4),
        sqrt(s.var5),sqrt(s.var6),sqrt(s.var7),sqrt(s.var8)),
        list(sqrt(m.var1),sqrt(m.var2),sqrt(m.var3),sqrt(m.var4),
        sqrt(m.var5),sqrt(m.var6),sqrt(m.var7),sqrt(m.var8)))
names(output) <- c("AA-ests of regression coefs",
        "Total Std. Error", "Samp. Std. Error of AA-ests",
        "Meas. Std. Error")
print(output)

##Compare to estimates and variances when using sample
##observations under deterministic measurement error:

##No measurement error:
summary(lm(y.omega ~ x.omega))$coefficients[,1:2]

##Berkson calibrated from means of x.rho8 (deterministic):
x.rho8 <- c(rep(NA,n))
for(i in 1:n){
                if(x.omega[i]<=5){x.rho8[i] <- x.l.trunc.mean}
                else if(x.omega[i] > 5){x.rho8[i] <- x.r.trunc.mean}
}
```

```
summary(lm(y.omega ~ x.rho8))$coefficients[,1:2]

##Classical calibration (deterministic) same (avg) error variance
##as RVVMs:
x.rho9 <- c(rep(NA,n))
for(i in 1:n){
                x.rho9[i] <- rnorm(1,x.omega[i],2)
}
summary(lm(y.omega ~ x.rho9))$coefficients[,1:2]
##

##Empirical approximation of the distributions of the OLS
##solutions (distributions over measurement uncertainty)
##for measurement protocol 4:
par(mfrow=c(1,2))
hist(coef4[,1],breaks=20,xlab="",
        main="Dist of sample OLS estimator for intercept")
hist(coef4[,2],breaks=20,xlab="",
        main="Dist of sample OLS estimator for slope")
```
