# Supplementary material for: Generalized measurement error: Intrinsic and incidental measurement error
Source: PLoS One. 2023 Jun 29;18(6):e0286680. doi: 10.1371/journal.pone.0286680 (PMC10309644; doi:10.1371/journal.pone.0286680)
Supplement: S3 File — (PDF) [file pone.0286680.s003.pdf]

S3 Appendix 3. Some selected generalized likelihoods for the toy dataset and proposed model of Example 10 follow.

Generalized likelihoods for the model $Y|X \sim N(\beta X, \sigma^2)$ using different sample values of $\rho_X(\omega_1)$, $\rho_X(\omega_2)$ for the toy dataset of Example 10 when $\sigma = 0.1$:

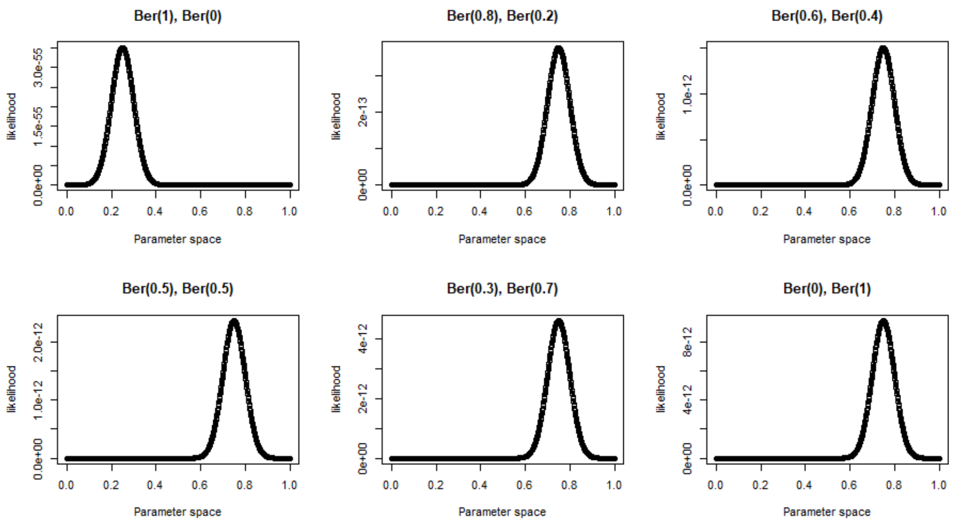

Generalized likelihoods for the model $Y|X \sim N(\beta X, \sigma^2)$ using different sample values of $\rho_X(\omega_1)$, $\rho_X(\omega_2)$ for the toy dataset of Example 10 when $\sigma = 0.4$:

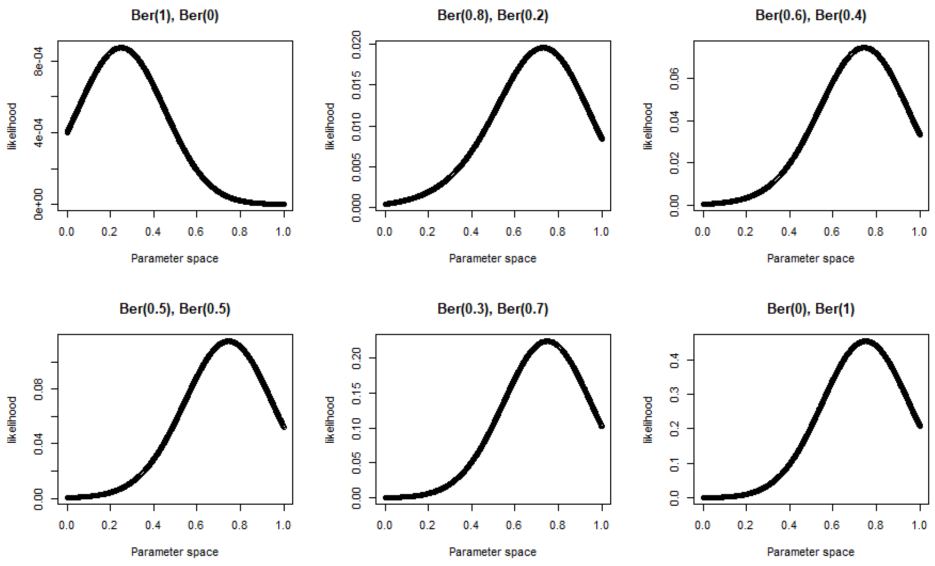

Generalized likelihoods for the model $Y|X \sim N(\beta X, \sigma^2)$ using different sample values of $\rho_X(\omega_1)$, $\rho_X(\omega_2)$ for the toy dataset of Example 10 when $\sigma = 0.5$:

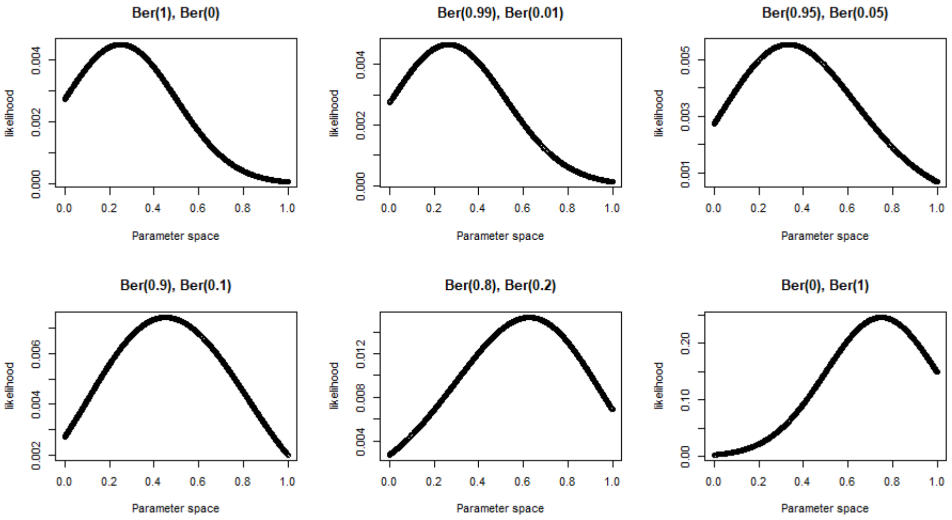

Generalized likelihoods for the model $Y|X \sim N(\beta X, \sigma^2)$ using different sample values of $\rho_X(\omega_1)$, $\rho_X(\omega_2)$ for the toy dataset of Example 10 when $\sigma = 1$:

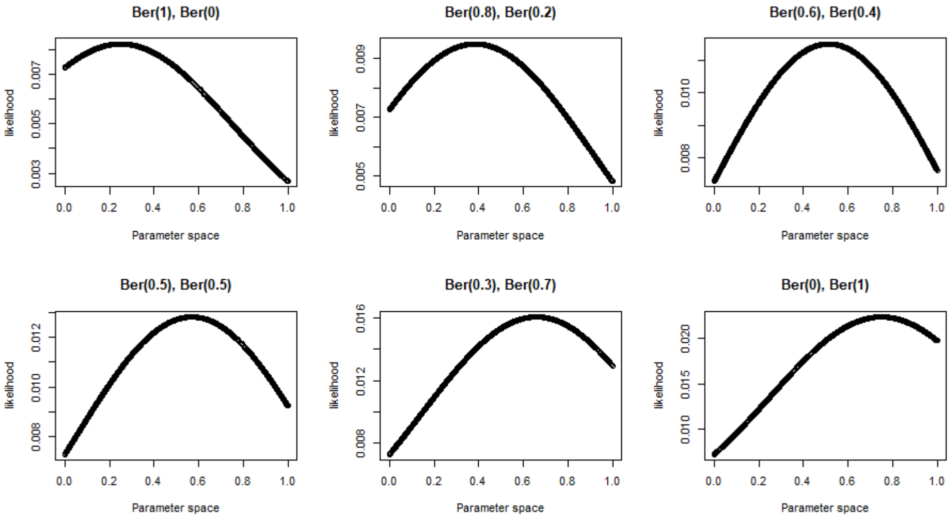

Generalized likelihoods for the model $Y|X \sim N(\beta X, \sigma^2)$ using different sample values of $\rho_X(\omega_1)$, $\rho_X(\omega_2)$ for the toy dataset of Example 10 when $\sigma = 5$:

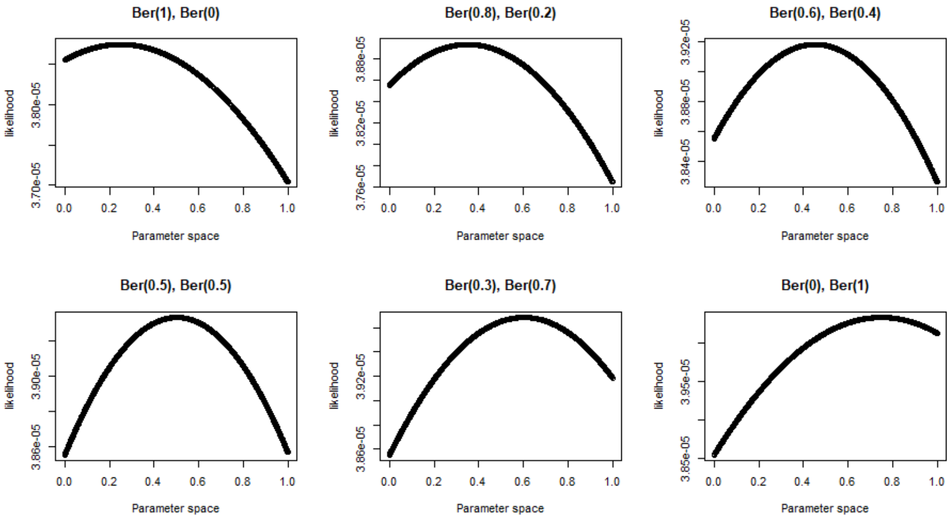
Ber(1), Ber(0)
likelihood
3.70e-05
3.80e-05
Parameter space
Ber(0.8), Ber(0.2)
3.76e-05
3.82e-05
3.88e-05
Ber(0.6), Ber(0.4)
3.84e-05
3.88e-05
3.92e-05
Ber(0.5), Ber(0.5)
3.86e-05
3.90e-05
Ber(0.3), Ber(0.7)
3.86e-05
3.92e-05
Ber(0), Ber(1)
3.85e-05
3.95e-05
0.0
0.2
0.4
0.6
0.8
1.0
