# Supplementary material for: Generalized measurement error: Intrinsic and incidental measurement error
Source: PLoS One. 2023 Jun 29;18(6):e0286680. doi: 10.1371/journal.pone.0286680 (PMC10309644; doi:10.1371/journal.pone.0286680)
Supplement: Attachment — Submitted filename: Revision.pdf [file pone.0286680.s004.pdf]

The paper presents a general notion of measuring errors, extending Berkson's work. The following manuscript does not present a particular measurement error model but provides measurement protocols and useful calibration methods. Indeed, it is challenging to collect experiment outputs adequately due to endogenous events characteristic of biodiverse systems presented as ``incidental measurement error" or errors produced by the measurement due to a suboptimal approach for collecting the results called ``intrinsic measurement error". From my point of view, the main interest of the presented method is the generalization of what can be meant by ``measurement error" under the framework of random-variable-value measurements (RVVMs). The content of the manuscript is useful. However, I have some minor observations/ questions to make before I recommend approval.

Minor Issues:

- I notice first and second section are connected to previous published work (Kroc [5] “Measurement protocols, random-variable valued measurements, and response process error: Estimation and inference when sample data are not deterministic”). For better clarity on the present manuscript, it needs to be clearly stated on the introduction. Note: On reference [5] remove the last point in the hyperlink, otherwise the URL is not found.
- The Berkson measurement error (BME) is not explicitly defined in the present manuscript.
- PLOS ONE template has the following scheme: abstract, introduction, materials and methods, results, discussion and conclusion. Some of the sections need to be renamed and presented in an appropriate order.
- Abstract: Review punctuation error before last sentence.
- Introduction: The paper starts presenting different examples about the different types of error emphasizing cases studied in mathematical psychology that are related with previous publication of the author, specially with citation[5]. It will be helpful to present a hyperlink to the full case for a better understanding of the case studies.
- Introduction: Define `` trivial” term specially for readers that have not a mathematical background
- Line 97: Do you want to said non-trivial?
- Discussion: The limitations and possible extensions of the present approach need to be clearly defined and explained. For example, is this generalization readily usable for fields other than mathematical psychology?
