# Supplementary material for: Generalized measurement error: Intrinsic and incidental measurement error
Source: PLoS One. 2023 Jun 29;18(6):e0286680. doi: 10.1371/journal.pone.0286680 (PMC10309644; doi:10.1371/journal.pone.0286680)
Supplement: Attachment — Submitted filename: review.pdf [file pone.0286680.s005.pdf]

Generalized measurement error: Intrinsic and incidental measurement error

This article generalizes the traditional notion of measurement error model by allowing the observed data to random valued. The idea that observed data can represent certainty/probability distribution instead of being real valued is interesting. As demonstrated by many examples, this framework could be useful for many applications. Generalized versions of the classical and Berkson error models are developed along with estimation strategies. Some numerical studies are conducted to investigate the performance of the proposed estimators. Given that this article does not contain any real data analysis the scope of numerical studies should be wider. Experiment with different scenarios. Particularly, studies designed to perform a comparison between the new framework and the traditional framework should be conducted.

1. A small literature review on the variety advances in measurement error framework would be useful for audience that is new to this area. Consider adding some references on measurement error involving different areas such as high dimensional data, bayesian framework and maybe even correlated data such as functional data. Many of these frameworks typically require additional data such as repeated measurements, instruments etc. Whereas the proposed framework seems to not require additional data. If this is indeed correct then highlighting this will add to the appeal of this work. Some references are suggested below.

    - Measurement Error in Nonlinear Models by D. Ruppert, Leonard A. Stefanski, David Ruppert
    - Functional measurement error in functional regression by S Jadhav, S Ma, Canadian journal of Statistics
    - A function-based approach to model the measurement error in wearable devices, S Jadhav, CD Tekwe, Y Luan, SIM
    - Covariate Selection in High-Dimensional Generalized Linear Models With Measurement Error, Journal of Computational and Graphical Statistics
    - Bayesian smoothing and regression splines for measurement error problems, SM Berry, RJ Carroll, D Ruppert, JASA

2. The notation $AA - \psi_\rho$ is confusing. It seems like it involves subtraction. Please reconsider the notation

3. The figures are not numbered. Only 3 figures are referred to in the text but there seem many more figures at the end.

4. Equation 5 is incorrect.

5. The simulation represented in equation 17 needs more clarity. For example, what is sample of observations? Is it $(\rho_{x^*}(\omega_i), \rho_y(\omega_i),)$? What is the true underlying regression equation?

6. In table 1, what are the true parameter values? Is $\beta_0 = 1, \beta_1 = 0.5$? If that is the case why are the intercept estimates more inaccurate compared to the slope estimate?

7. Discuss this results in Table 1 in more detail. Do you expect any differences in the results between Berkson calibrated and classically calibrated cases? Do the results match? If no then, why not?

8. Lines 812-814 say that attenuation for slope is stronger for Berkson absent case. But results for all the three cases seem similar.

9. More numerical exploration will add more insights to the proposed framework. Some suggestions a) another example as 9, but with a non-normal distribution such as uniform, b) regression set-up but response also has measurement error

10. A natural question is how does the proposed framework compare with the classical framework? You can simulate some data mimicking some realistic scenarios (these could be the motivating examples given earlier in the manuscript) and compare performance of the two frameworks. For example: Suppode the aim is to determine the effect of X on Y in a regression set up. Generate data set with X as binary covariates and another where protocol is binomial distribution. Use the proposed estimation method on the binomial protocol and a traditional measurement error correction based methods for binary case to estimate the slope.

11. To better show the behaviour as $\sigma \to 0$ add smaller values of σ such as 0.1, 0.05 etc to Table 2.
