# Supplementary material for: Generalized measurement error: Intrinsic and incidental measurement error
Source: PLoS One. 2023 Jun 29;18(6):e0286680. doi: 10.1371/journal.pone.0286680 (PMC10309644; doi:10.1371/journal.pone.0286680)
Supplement: Attachment — Submitted filename: review- pone-d-22-29796_r1.pdf [file pone.0286680.s006.pdf]

There is a large body of literature surrounding measurement error, its impact, and how to correct for it in the “classical” sense that the author seeks to generalize. However, this author calls this type of measurement error “inessential.” Plenty of others have shown the bias and variance inflation attributable to ignoring this type of measurement error, so I cannot see how it would not be essential to correct for it. I am generally uneasy about the paper’s treatment of the established measurement error literature and feel that it detracted from the overall message.

For readers like myself who are accustomed to “incidental” measurement error, I think further discussion and nuance is needed to first differentiate between the two types being proposed. Some of the points currently used to distinguish between them I do not agree with as written. For example, “intrinsic” measurement error is said be observation-specific, but existing approaches to “incidental” measurement error can allow for this (perhaps to a different degree), too. Without a clear distinction between the types, it can be hard to follow the rest of the paper and see the larger contributions of the proposed generalized framework.

Some other points for the author’s consideration are below.

- If much of the literature focuses on real numbers, is it not because most applications involve real numbers? To understand the importance of this manuscript’s extension to non-real numbers, I think some justification is needed for why this hasn’t been handled in the past but is needed. Also, the first three examples given all seem to have real-valued measurements.
- “Measurement uncertainty is allowed to be potentially unique to each element.” I often see methods and applications in the literature where measurement error is allowed to depend on other factors (e.g., study site or machine type). I don’t see how this (“unique to each element”) differentiates the author’s two types of measurement error.
- In Equation (2), I expected the decomposition to be $f(x^*,x) = f(x|x^*)f(x^*)$, since it would only require modeling $f(x|x^*)$ since $f(x^*)$ is fully observed.
- In Equation (3), Y and Z have not yet been defined.
- The way the names for the types of measurement error are introduced (e.g., “intrinsic or essential”), to me, imply that the two (“intrinsic” and “essential”) names are synonymous, but they are not.
- Why is the classical measurement error model introduced in Section 1 but Berkson not (since it will be revisited later)?
- In the sentence after Equation (6) there is an i.e., inside of an i.e.,.
- Though I appreciate the many examples, this is a pretty lengthy paper; I think only one or two examples should be kept in the main text and perhaps the rest moved to the supplement.
- R code should be included as an R script (a .R file), rather than printed in a PDF Supplement. It is cumbersome for readers to copy and paste code from a PDF, and also can be prone to errors. The ready-to-run code for the paper should be available online, for example, on figshare or GitHub.
