# Supplementary material for: Generalized measurement error: Intrinsic and incidental measurement error
Source: PLoS One. 2023 Jun 29;18(6):e0286680. doi: 10.1371/journal.pone.0286680 (PMC10309644; doi:10.1371/journal.pone.0286680)
Supplement: Attachment — Submitted filename: Response to Reviewers.pdf [file pone.0286680.s007.pdf]

May 3, 2023

Edward Kroc
Measurement, Evaluation, and Research Methodology
University of British Columbia
2125 Main Mall, Vancouver, BC, Canada, V6T1Z4
Phone: 1-604-822-8671, Email: ed.kroc@ubc.ca

Response to the Editor and Reviewers

Regarding manuscript PONE-D-22-29796,
Generalized measurement error: Intrinsic and incidental measurement error

Responses to the Editor

I'd like to start by thanking the editor for the handling of this manuscript and for eliciting feedback from reviewers in a timely manner. I respond to each of the reviewers in detail below. All rewritten pieces of the manuscript have been highlighted in yellow in the attached marked-up copy. Regarding the points raised by the Editor:

1. *The reviewers' comments highlighted that a detailed discussion of the current state-of-the-art should be done.*
    - **A review has now been included in the Introduction section, supplemented by a further review of connected ideas in the Discussion section. See my response to Comment 2 from Reviewer 4.**
2. *In addition, the architecture of the proposed model needs to be shown and described in detail.*
    - **This has been done mathematically in the Methods section in detail, but I have now added extended examples illustrating the technique and contrasting it with current approaches in the Results section. See my responses to Comments 1 and 6 through 10 from Reviewer 4. Additionally, I have now clarified in the Introduction how the framework proposed in this paper is intended to operate informally (the formal details appear in the Methods section). See my responses to Comments 2, 3, and 4 from Reviewer 5.**
3. *They also pointed out that spell-checking and typo proofreading of the manuscript are required.*
    - **The manuscript has now been proofread and edited for grammatical content.**

Responses to Reviewer 2

1. *The manuscript has been thoroughly revised. I suggest a re-read and improvement of the english form.*

    - **Many thanks. The manuscript has been further proofread and edited for grammatical content.**

Responses to Reviewer 4

1. *This article generalizes the traditional notion of measurement error model by allowing the observed data to random valued. The idea that observed data can represent certainty/probability distribution instead of being real valued is interesting. As demonstrated by many examples, this framework could be useful for many applications. Generalized versions of the classical and Berkson error models are developed along with estimation strategies. Some numerical studies are conducted to investigate the performance of the proposed estimators. Given that this article does not contain any real data analysis the scope of numerical studies should be wider. Experiment with different scenarios. Particularly, studies designed to perform a comparison between the new framework and the traditional framework should be conducted.*

    - **Thanks for these comments and suggestions. I have now expanded the scope of some of the numerical studies in this paper to supplement the theoretical treatment. Originally, Examples 1, 2, and 7 contrasted the situations between the generalized framework of RVVMs and the classical framework of measurement error arising from deterministic sample measurements. However, I have now greatly expanded Example 9 to incorporate a wide range of comparisons. In this example, I now contrast 11 different measurement protocols for studying a population-level relationship between a response and predictor in the ordinary linear regression context. Three of these measurement protocols are deterministic, one representing the case of Berkson error in the predictor, one polluted by classical measurement error, and one free of all measurement error. Another 6 measurement protocols explore what happens under various calibration conditions for general RVVMs, some first or second order Berkson calibrated, some classically calibrated, some both. The final 2 example measurement protocols explore what happens when the response variable is polluted with intrinsic measurement error. I take the opportunity to also contrast what happens to the attenuation factors for the OLS estimate of the simple regression slope in these different cases. Please see the revised manuscript for all the details. Additionally, I have expanded and clarified the comparisons between the classical and RVVM framework in Example 10.**

2. *A small literature review on the variety advances in measurement error framework would be useful for audience that is new to this area. Consider adding some references on measurement error involving different areas such as high dimensional*

data, bayesian framework and maybe even correlated data such as functional data. Many of these frameworks typically require additional data such as repeated measurements, instruments etc. Whereas the proposed framework seems to not require additional data. If this is indeed correct then highlighting this will add to the appeal of this work.

- **This is a good idea. I have now included a brief literature review early on in the Introduction to emphasize the fact that all measurement error techniques involve some combination of additional data and/or statistical models relating the target measurand to an error-prone proxy. I clarify up-front here too that the framework of RVVMs *does* require additional data, but that it does *not* necessarily require an analyst to propose a statistical model relating the measurand to its error-prone observable. The two new paragraphs read:**

> All treatments of measurement error invariably require additional data and/or a statistical model to relate the target quantity of interest X to its observable and error-prone proxy X^*. For instance, in a validation study, the error-free values of X are observed for a subsample. These can then be used to validate a proposed measurement error model relating X to X^* improving inferences dependent on X when using only the observed values of X^* on the entire sample [10, 11]. Replication studies require two or more error-prone proxies for X which can then be combined to improve inferences dependent on X via some kind of statistical model [12, 13, 14]. Notably, this paradigm is the one most commonly employed in factor analysis for the study of latent variables, ubiquitous in the scale development and survey literature of the behavioural sciences [15, 16, 17]. Calibration studies extend this idea to when some error-prone proxy is subject to systematic or differential measurement error, usually requiring a more complex regression framework to relate the various error-prone proxies to the true measurand of interest, perhaps dependent on other exogenous sources of information that could be measured with or without measurement error [18, 19].
>
> The framework of RVVMs also requires additional data, but it does not necessarily require the specification of a statistical model relating the error-prone sample observations to the target measurand of interest. Specifically, to use RVVMs in a nontrivial way, we require additional information about the *confidence* or *certainty* that the sample measurement assumes a set of values. This additional infor-

mation must be supplied by the measurement apparatus, as when a respondent answering a survey item tells us their subjective level of confidence in their response, say on a 0-100% scale. This extra "confidence information" is then combined with their point response (e.g., their chosen response to the survey item) to define a real-valued random variable. Typically, the point response may define this RVVM's mean and the confidence information may be used to define its variance. In some situations, this will require assuming a parametric distributional structure for the RVVM (so a statistical model), but in other situations the probabilistic structure will be automatically specified by the combination of sample data, in particular when the measurand is a categorical quantity. RVVMs thus require more information from the measurement apparatus to improve estimates and inferences, rather than necessarily requiring an analyst to propose a measurement error model.

3. *The notation $AA - \psi_\rho$ is confusing. It seems like it involves subtraction. Please reconsider the notation*

   - **This is a fair point. I have changed the notation throughout the manuscript to a functional form: $AA(\psi_\rho)$.**

4. *The figures are not numbered. Only 3 figures are referred to in the text but there seem to be many more figures at the end.*

   - **The numbering has been checked for all figures and should now be in order. I have added a line referring the interested reader to the figures that appear in Supplemental information S3; this direction was previously missing.**

5. *Equation 5 is incorrect.*

   - **I believe the reviewer is mistaken here. What distinguishes the Berkson error structure from the classical error structure is only the conditional expectation condition, $\mathbb{E}(E \mid X^*) = 0$ for the former and $\mathbb{E}(E \mid X) = 0$ for the latter (these conditions are sometimes replaced by stronger independence assumptions). Writing $X^* = X + E$ or $X = X^* + E$ is immaterial (they are equivalent up to a minus sign on the error, which is immaterial since one can always redefine the arbtirary notation $E' = -E$). See, for instance, Carroll et al. (2006) p.5, or Yi et al. (2021) p.49.**

6. *The simulation represented in equation 17 needs more clarity. For example, what is sample of observations? Is it* $(\rho_{x^*}(\omega_i), \rho_y(\omega_i))$*? What is the true underlying regression equation?*

   - **This simulation example has now been greatly expanded and restructured for clarity. The revised text is long, so please see the revised manuscript for these changes. Briefly though, in regards to the reviewer's questions here, the notation for the measurement protocols has been clarified and enumerated in a new table (Table 1), and the underlying population level relationship and the regression equation used to estimate it have both been given stand-alone equation numbers to make them stand out, and also to make referring to them later easier to do.**

7. *In table 1, what are the true parameter values? Is* $\beta_0 = 1$, $\beta_1 = 0.5$*? If that is the case why are the intercept estimates more inaccurate compared to the slope estimate?*

   - **Yes, Table 1 (now Table 2 in the revised manuscript) contains the model estimates using the simple regression model specified in equation (16) for the underlying population-level relationship between X and Y given in lines 786-87 of the original manuscript; thus, $\beta_0 = 1$ and $\beta_1 = 0.5$. This has been clarified in the table's caption and I have given the underlying population-level relationship its own equation label to make it easier to refer to in the text (the value for β_1 has also changed from 0.5 to 5; see my response to Comment 9 for the reason why). The sample estimates for the intercept are more inaccurate than the slope estimates for all measurement protocols (including the one free of intrinsic measurement error) because the sample data are generated to have support that fall mostly away from the intercept at $X = 0$; this is expected for any regression analysis where the range of the predictor data fall away from the response intercept and one has not centered the predictors. I have pointed this out in the updated text.**

8. *Discuss this results in Table 1 in more detail. Do you expect any differences in the results between Berkson calibrated and classically calibrated cases? Do the results match? If no then, why not?*

   - **Please see the revised manuscript for the added exposition addressing this criticism. To explain many of the differences between the model estimates based on the various measurement protocols, I now**

make use of the classical idea of attenuation factors for a simple regression slope. This adds a bit of mathematics that is discussed briefly in the text, but I have also added detailed derivations of these quantities in Supplemental information S1.

9. *Lines 812-814 say that attenuation for slope is stronger for Berkson absent case. But results for all the three cases seem similar.*

   - **I have changed the population-level relationship between X and Y now to make the differences in attenuation easier to see. Previously, the population-level slope was set at 0.5 but it is now set at 5. Moreover, I've added theoretical derivations of the attenuation factors for the various measurement protocols to better illustrate the differences across the different measurement scenarios. These mathematical details appear in Supplemental information S1.**

10. *More numerical exploration will add more insights to the proposed framework. Some suggestions a) another example as 9, but with a non-normal distribution such as uniform, b) regression set-up but response also has measurement error.*

    - **This is a good suggestion. I have added 8 new measurement protocols to Example 9 and streamlined the corresponding simulation code in Supplemental Information S2. The new measurement protocols explore differences when the RVVMs are normal or uniform distributed, first and second order Berkson calibrated, first order classically calibrated, and when the response variable is also subject to intrinsic measurement error.**

11. *A natural question is how does the proposed framework compare with the classical framework? You can simulate some data mimicking some realistic scenarios (these could be the motivating examples given earlier in the manuscript) and compare performance of the two frameworks. For example: Suppose the aim is to determine the effect of X on Y in a regression set up. Generate data set with X as binary covariates and another where protocol is binomial distribution. Use the proposed estimation method on the binomial protocol and a traditional measurement error correction based methods for binary case to estimate the slope.*

    - **This is a good suggestion and it has now been integrated into Example 10. Please see the revised manuscript for the new exposition.**

12. *To better show the behaviour as $\sigma \to 0$ add smaller values of σ such as 0.1, 0.05 etc to Table 2.*

- **I have added a column to this table (now Table 3) for when $\sigma = 0.3$. Even at this large a value for σ, all but one of the slope estimates have stabilized to the deterministic upper boundary case of $\widehat{\beta} = 0.75$.**

Response to Reviewer 5

1. *There is a large body of literature surrounding measurement error, its impact, and how to correct for it in the "classical" sense that the author seeks to generalize. However, this author calls this type of measurement error "inessential." Plenty of others have shown the bias and variance inflation attributable to ignoring this type of measurement error, so I cannot see how it would not be essential to correct for it. I am generally uneasy about the paper's treatment of the established measurement error literature and feel that it detracted from the overall message.*

   - **I am thankful that a reviewer weighed in on the descriptive terminology I have proposed in this paper. I admit that I too have always felt a bit uneasy about distinguishing these two types of measurement error as "essential" and "inessential," though I do think the terminology makes sense from a purely mathematical point of view (i.e., one type, "inessential," is generated by trivial probability distributions, point mass measures, while the other, "essential," is generated by nontrivial distributions). I think the terminology of "incidental" and "intrinsic" do an equally sufficient job of mathematically distinguishing the two types of error, but don't have quite the same judgmental implications in a general context. I have therefore removed the "inessential/essential" terminology throughout the paper and used only the "incidental/intrinsic" descriptors, as in the title of the paper.**

2. *For readers like myself who are accustomed to "incidental" measurement error, I think further discussion and nuance is needed to first differentiate between the two types being proposed. Some of the points currently used to distinguish between them I do not agree with as written. For example, "intrinsic" measurement error is said be observation-specific, but existing approaches to "incidental" measurement error can allow for this (perhaps to a different degree), too. Without a clear distinction between the types, it can be hard to follow the rest of the paper and see the larger contributions of the proposed generalized framework.*

   - **To my knowledge, all current approaches to measurement error require some kind of exchangability of sample measurements over a subset of the sample space. Generally, one sees relationships like the likelihood specification of equation (3). Notably, the measurement model is used to relate the noisy proxy x^* to the true measurand of interest x and (perhaps) other variables y, z via the conditional density function $f(x^*|y, x, z)$. But this defines a probability structure for the error-prone proxy x^* that is conditional**

on the values of these other random variables. This implies that for any fixed values of those conditioning variables, the probability action is the same.

Many different sample units in the sample space can generate the same values of the conditioning variables, and this necessarily leads to the same probability specification (i.e., the same probability density function) for $f(x^*|y, x, z)$. For example, the random quantities y, x, and z can be constant on an arbitrarily large subset $\mathcal{S}$ of the overall sample space Ω, and this then forces the conditional density function for x^* to be an unchanging function on $\mathbb{R}$ over $\mathcal{S}$.

The framework of RVVMs proposed in this paper is different because the probability action of a noisy proxy for x is allowed to be potentially different *at every element of the sample space* Ω. One uses extra information (i.e., the uncertainty encoded in the RVVMs) to possibly allow the conditional density $f(x^*|y, x, z)$ to completely change from one sample element to the next, even if the conditioning variables y, x, and z are constant (and one does not need to condition on any auxilliary variables at all with RVVMs).

Essentially, RVVMs allow one to *condition on the individual sample element*, rather than only on the values of other random variables (observed or not). This necessitates the use of measure theory throughout the paper, because one can no longer make sense of a quantity like $f(x^*|y, x, z)$ in general. Instead, one could talk about the quantity $f(x^*|\omega)$ for $\omega \in \Omega$ (though making sense of such a quantity requires some care, as unpacked in the Methods section). Fixing $\omega \in \Omega$ would automatically fix the values of any ancilliary random variables like x, y, and z, so there is no longer any need to condition on them. But now one can distinguish between two different probability actions $f(x^*|\omega)$ and $f(x^*|\omega')$ for $\omega \neq \omega' \in \Omega$, even if x, y, and z are unchanged from ω to ω'. Because of this finer granularity, RVVMs turn measurement error into a data collection problem (i.e., each sample observation needs to give us confidence/certainty information) rather than a modelling problem (i.e., needing a statistical model to relate a noisy proxy to the true measurand). I have not been able to find any way to encode such sample-unit-specific information about a measurement using the classical framework of measurement error theory. I have tried to make this distinction more clear in the Introduction of the paper now, though full details are still deferred to the Methods section. I have added the following lines to the Introduction:

Specifically, intrinsic measurement error allows one to encode measurement uncertainty at the level of a single sample unit within a sample space. Informally, one can imagine replacing the measurement model $f(x^*|y, x, z)$ of eq (3) by one that looks like $f(x^*|\omega)$ for an arbitrary $\omega \in \Omega$. The probability action of the error-prone observable for x can thus be unique to each sample unit in the sample space, rather than only unique to a subset of the sample space defined by a fibre of the conditioning variables x, y, and z. This has the effect of converting a modelling problem into a problem of data collection.

3. *If much of the literature focuses on real numbers, is it not because most applications involve real numbers? To understand the importance of this manuscript's extension to non-real numbers, I think some justification is needed for why this hasn't been handled in the past but is needed. Also, the first three examples given all seem to have real-valued measurements.*

   - **The framework of RVVMs is designed to handle measurements for real-valued quantities of interest, just as the classical measurement error theory is. As the reviewer mentions, the first three examples all describe real-valued measurands. Classically, one observes an error-prone proxy for the measurand of interest that is a real-valued random variable; thus, a sample measurement at $\omega \in \Omega$ corresponds to a single real number $X^*(\omega)$. Using RVVMs however, the error-prone proxy is encoded as a probability measure on the reals. Now for a sample measurement at $\omega \in \Omega$, the observed measurement $\rho_X(\omega)$ describes all the possible values for the observable, with each possible value weighted according to the definition of the measure. For trivial RVVMs, this measure is just a point mass and thus defines a random variable that is equal to $X^*(\omega)$ with probability 1. But in general, this measure encodes the certainty that the sample measurement is equal to any of many different real values with varying probabilities.**

     Critically, the targets of measurement are still real-valued quantities; RVVMs do not propose studying different types of measurands. But this paper works out the mathematics that allows one to take a probability measure on the reals as an observable quantity and relate it to a real-valued measurand of interest (see the Methods section). I have added a few words to the Abstract of the paper to try to clarify this:

     In this paper, we generalize the notion of measurement error for a

> real-valued measurand on deterministic sample datasets to accommodate sample data that are random-variable-valued. This leads to the formulation of two distinct kinds of measurement error: *intrinsic* measurement error, and *incidental* measurement error. Incidental measurement error will be recognized as the traditional kind that arises from a set of deterministic sample measurements, and upon which the traditional measurement error modelling literature is based, while intrinsic measurement error reflects some subjective quality of either the measurement tool or the real-valued measurand itself.

And also in the Introduction:

> What we relax in what follows is that we do not require these sample data to form a set a real numbers. Instead, we will consider a generic *measurement protocol* ρ_X for a real-valued target (random) phenomenon of interest that is applied to each of the sampled units $\omega_1, \ldots, \omega_n$.

4. *"Measurement uncertainty is allowed to be potentially unique to each element." I often see methods and applications in the literature where measurement error is allowed to depend on other factors (e.g., study site or machine type). I don't see how this ("unique to each element") differentiates the author's two types of measurement error.*

    - **As explained in my above response to Comment (2), RVVMs allow one to specify potentially unique uncertainty in a sample measurement *for every element of a sample space.* In the customary approach to measurement error, one can only accommodate uncertainty in the sample measurement conditional on other factors, like study site or machine type. If these other factors are categorical in nature, then one needs multiple observations on these background factors in order to estimate any model-based relationship between the observable noisy proxy and the measurand of interest (via a measurement error model), and if they are continuous than stronger modelling assumptions are required (e.g., continuity on average between observed values). None of this is strictly necessary in the RVVM framework because how the uncertainty in the sample measurements relates to the measurand can be described by the different kind of sample data one collects (i.e., not just a point observation, but also some kind of information about how potential point observations might be distributed for the particular sample unit). Mathematically, this boils down to the difference of being**

able to condition on a single unit of the sample space (RVVMs) vs. only on a level set of some other factors (traditional approaches to measurement error).

5. *In Equation (2), I expected the decomposition to be* $f(x^*, x) = f(x|x^*)f(x^*)$*, since it would only require modeling* $f(x|x^*)$ *since* $f(x^*)$ *is fully observed.*

   - **What the reviewer has described is a typical likelihood specification for a Berkson measurement error problem, where one conditions on the observations and then proposes a model as to how the true (unobserved) values of the target of measurement,** x**, vary around the observed proxy,** x^***. The more common specification (at least in the classical literature) imposes a classical (Pearson/Spearman) measurement error structure instead, and this is what I have described in Equation 2. See, for example, Chapter 4 of Gustafson (2003). Importantly, both conditional density decompositions are valid and potentially useful, but if one wants to impose a certain kind of calibration structure (classical or Berkson) on the measurement problem then one decomposition is much more useful than the other. I have included both decompositions in the definition of Equation (2) now and clarified when one is more useful than the other:**

     > In applied modern practice, the algebraic relation in (1) is often replaced by a likelihood specification, such as
     >
     > $$f(x, x^*) = f(x^*|x)f(x), \text{ or } f(x, x^*) = f(x|x^*)f(x^*). \tag{2}$$
     >
     > Here, $f(x, x^*)$ is the joint density of X and X^* decomposed into the usual product of conditional and marginal densities. The former decomposition is typically used when assuming classically calibrated measurement errors and the latter decomposition when assuming Berkson calibrated errors [2].

6. *In Equation (3),* Y *and* Z *have not yet been defined.*

   - **Thanks for catching this. I have now clarified that** Y **is a response variable and** Z **is a set of predictor variables free of measurement error. The following line has been added:**

     > In practice, one may be interested in building some kind of regression model for a response Y dependent on a predictor X measured with measurement error and on other predictors Z that are free of

measurement error. Let X^* be the noisy measurement for X. In this framework, one generally sees specifications like eq (3).

7. *The way the names for the types of measurement error are introduced (e.g., "intrinsic or essential"), to me, imply that the two ("intrinsic" and "essential") names are synonymous, but they are not.*

    - **Yes, I was using the terms "intrinsic/essential" and "incidental/inessential" both interchangably, but as noted above, I agree with the reviewer that the "essential/inessential" terminology is too potentially misleading. Thus, it has been eliminated.**

8. *Why is the classical measurement error model introduced in Section 1 but Berkson not (since it will be revisited later)?*

    - **I have now introduced both in Equation (2) of Section 1. See the response to Comment 5.**

9. *In the sentence after Equation (6) there is an i.e., inside of an i.e.,.*

    - **This has now been fixed: I have eliminated the second "i.e."**

10. *Though I appreciate the many examples, this is a pretty lengthy paper; I think only one or two examples should be kept in the main text and perhaps the rest moved to the supplement.*

    - **The many examples were mostly added to satisfy the other reviewers of this manuscript that wanted to see more examples in the paper. I appreciate that the reviewer may prefer fewer in the body of the text, but other reviewers seemed to prefer more. I am inclined to stick with the examples that I have in the text as they currently appear as they all illustrate important theoretical points and/or address specific questions brought up during peer review. The examples have also been numbered so a reader can easily identify them and skip them if they are so inclined.**

11. *R code should be included as an R script (a .R file), rather than printed in a PDF Supplement. It is cumbersome for readers to copy and paste code from a PDF, and also can be prone to errors. The ready-to-run code for the paper should be available online, for example, on figshare or GitHub.*

    - **Yes, this is a good idea. I have now uploaded the code to GitHub:** `https://github.com/edkroc/Supplemental_information_S2_PLOSOne_`

`GeneralizedMeasurementError2023`**, and directed the reader to this online location in the PDF version of the Supplemental information S2 file.**
